# Supplementary material for: Feasibility of Utilizing Stable-Isotope Dimethyl Labeling in Liquid Chromatography–Tandem Mass Spectrometry-Based Determination for Food Allergens—Case of Kiwifruit
Source: Molecules. 2019 May 18;24(10):1920. doi: 10.3390/molecules24101920 (PMC6571768; doi:10.3390/molecules24101920)

**Supplementary Table S1.** Identified allergens of the kiwi extracts.

| Protein ID | IUIS name | Protein name | MASCOT score | Coverage |
|---|---|---|---|---|
| P00758 | Act d 1 | Actinidian | 1577 | 47.9 |
| P81370 | Act d 2 | Thaumatin-like protein | 906 | 60.9 |
| Q6TPK4 | Act d 4 | Cysteine proteinase inhibitor 1 | 287 | 71.4 |
| P84527 | Act d 5 | Kiwellin | 1469 | 42.7 |
| P83326 | Act d 6 | Pectinesterase inhibitor | 511 | 26.2 |
| P85076 | Act d 7 | Pectinesterase | 320 | 14.1 |
| P86137 | Act d 10 | Non-specific lipid-transfer protein 1 | 41 | 14.1 |
| P85524 | Act d 11 | Kirola | 437 | 58.7 |

**Supplementary Table S2**. BLAST results of candidate peptides with 100% identity and 100% query coverage

| Protein | Peptide sequence | Protein in non-*Actinidia* species (species) | Protein ID |
|---|---|---|---|
| Act d 1 | SAGAVVDIK | —[a] | — |
| Act d 5 | NNIVDGSNAVWSALGLDK | — | — |
| | IVALSTGWYNGGSR | Ripening-related protein grip22 (Vitis vinifera) | Q9M4H4 |
| | | Uncharacterized protein (Citrus unshiu) | A0A2H5Q537 |
| | | Uncharacterized protein (Vitis vinifera) | F6GU22 |
| | | Grip22-like protein (Vitis quinquangularis) | A0A163HAW5 |
| | | Uncharacterized protein (Cucumis sativus) | A0A0A0KBF2 |
| | | Uncharacterized protein (Cucumis sativus) | A0A0A0KGH1 |
| | | Kiwellin-like (Cucumis melo) | A0A1S3CI58 |
| | | Uncharacterized protein (Citrus clementina) | V4SG37 |
| | | Ripening-related protein (Siraitia grosvenorii) | K7NBR3 |
| | | Uncharacterized protein (Gossypium barbadense) | A0A2P5Q249 |
| | | Uncharacterized protein (Cucumis sativus) | A0A0A0KDC3 |
| | | Kiwellin-like (Gossypium hirsutum) | A0A1U8J808 |
| | | kiwellin-like (Cucumis melo) | A0A1S3CNR1 |
| | | Uncharacterized protein (Cucumis sativus) | A0A0A0K532 |
| Act d 11 | GEHNSVTWTFHYEK | — | — |

[a]The query peptide matched to no proteins with 100% identity and 100% coverage after the BLAST search

**Supplementary Fig S1**. Mass spectra of (A) SPA5-H, (B) SPA5-D, (C) SPA11-H and (D) SPA11-D. The dotted line represented the m/z of native signature peptides (SPA5 and SPA11) and Δm/z showed the m/z shift of the signature peptide before and after stable-isotope dimethyl labeling.

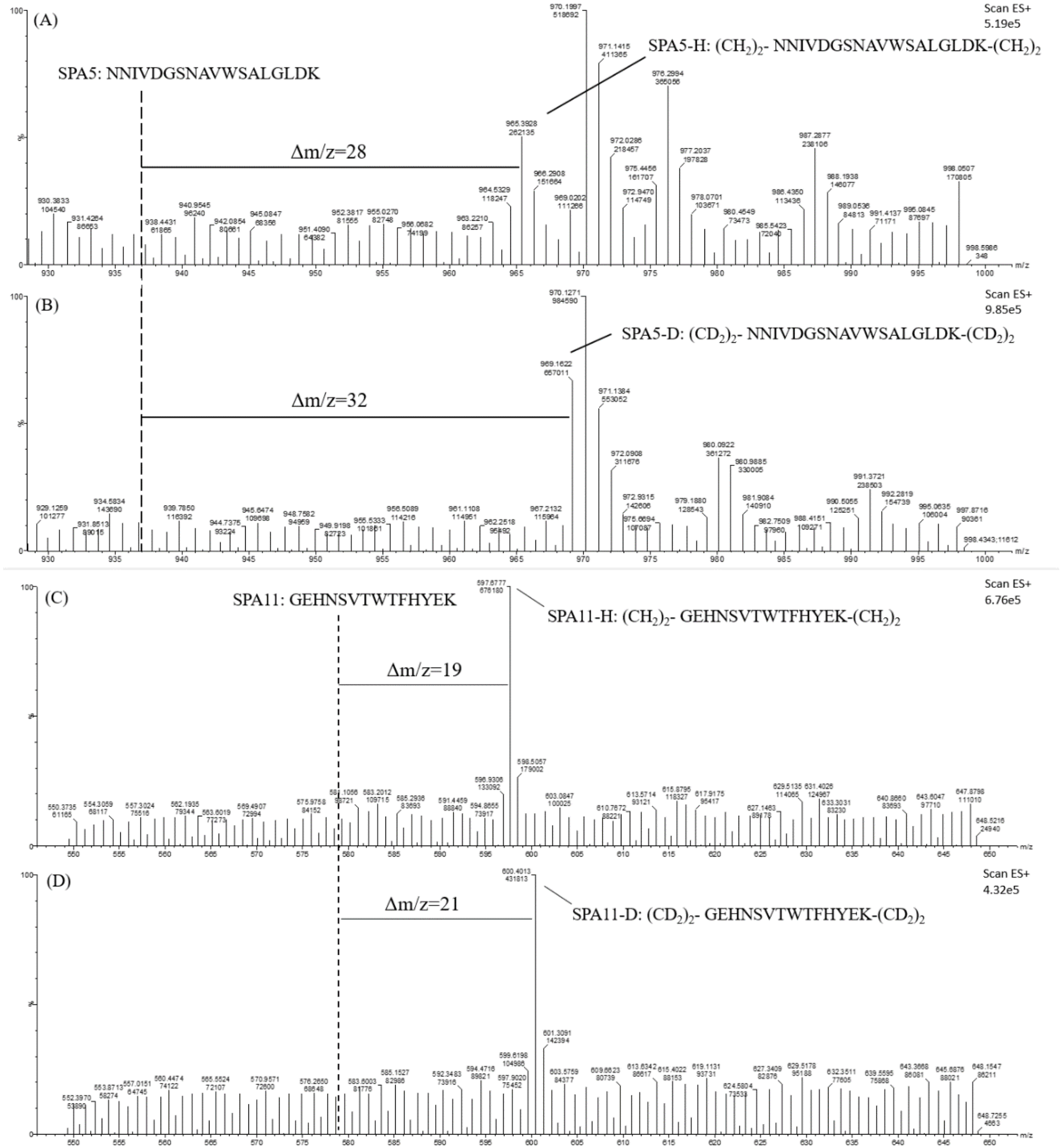

**Supplementary Fig. S2** MRM chromatograms of (A) SPA5-H (1 μg $mL^{-1}$), (B) SPA5-D (500 ng $mL^{-1}$), (C) SPA11-H (1 μg $mL^{-1}$), and (D) SPA11-D (500 ng $mL^{-1}$) for the mixture of peptide standards and (E) SPA5-H (568.2 μg $mL^{-1}$), (F) SPA5-D (500 ng $mL^{-1}$), (G) SPA11-H (no peak detected), and (H) SPA11-D (500 ng $mL^{-1}$) for kiwifruit raw extract sample.

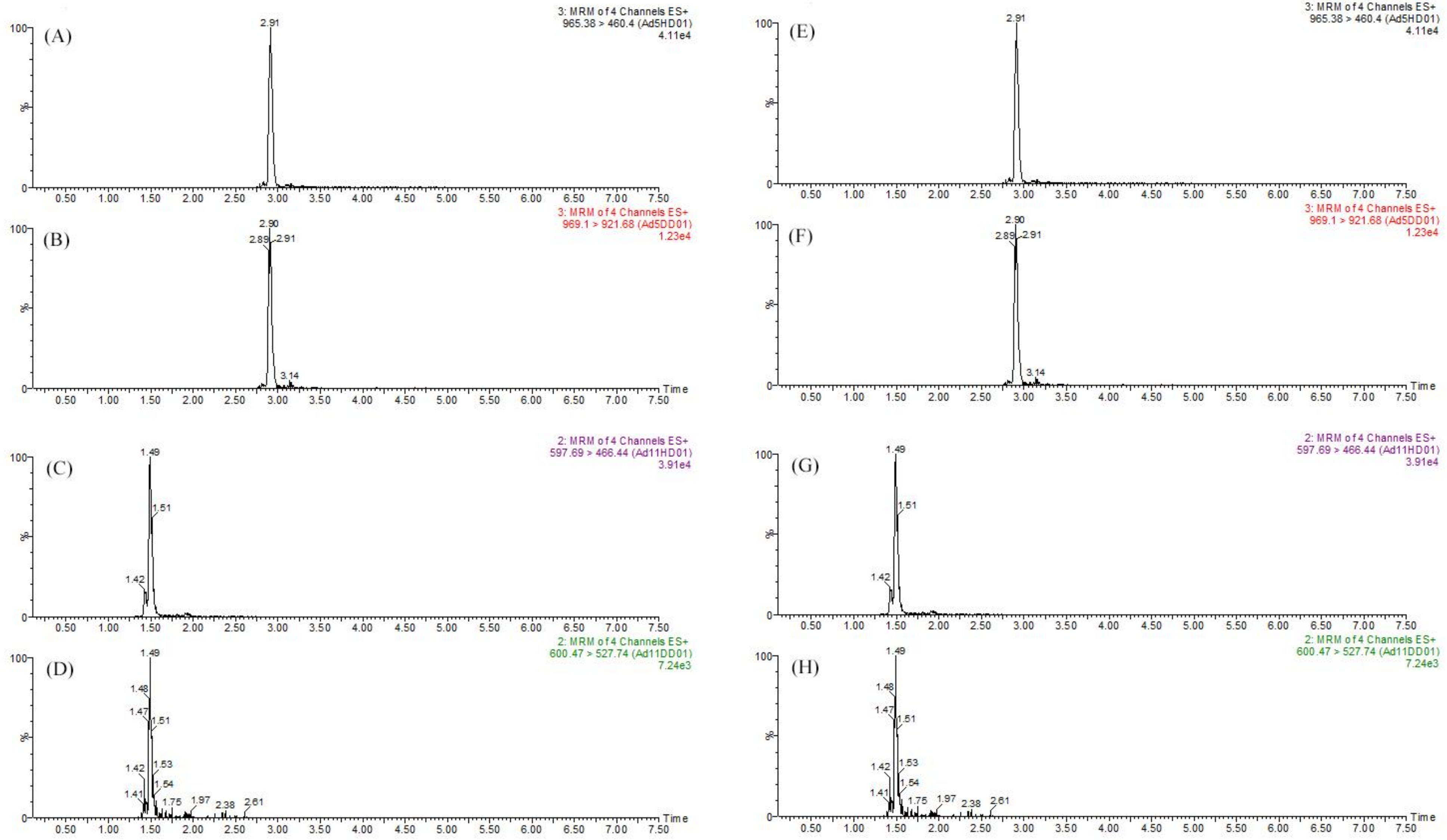

**Supplementary Fig. S3** Comparison of phenol extraction efficiency.

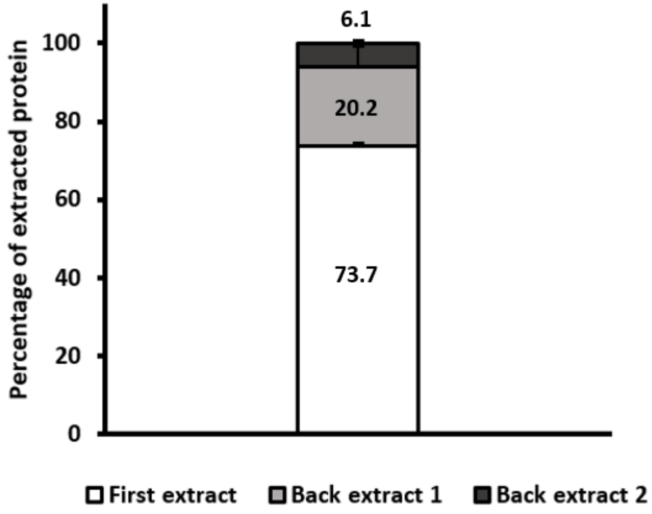

**Supplementary Fig. S4** Comparison of the extraction efficiency of phenol method from reference and the modified phenol method.

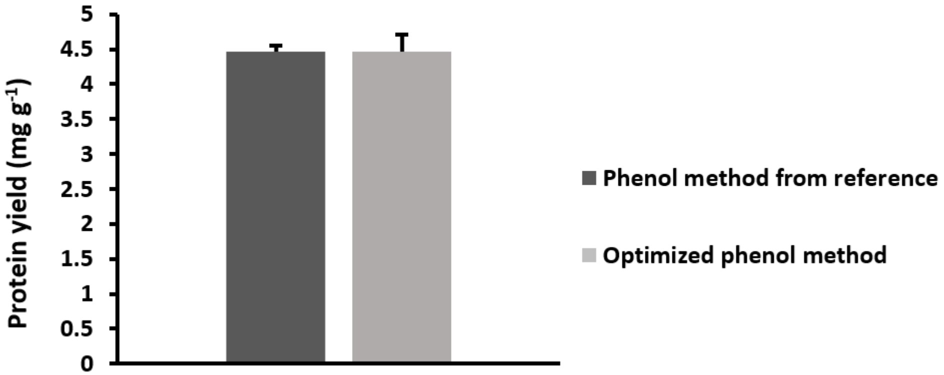

Supplement: Supplementary file 1 [file molecules-24-01920-s001.pdf]
